# New Functional Foods with Cactus Components: Sustainable Perspectives and Future Trends

**DOI:** 10.3390/foods12132494

**Published:** 2023-06-27

**Authors:** Shênia Santos Monteiro, Raphael Lucas Almeida, Newton Carlos Santos, Emmanuel Moreira Pereira, Amanda Priscila Silva, Hugo Miguel Lisboa Oliveira, Matheus Augusto de Bittencourt Pasquali

**Affiliations:** 1 Post-Graduate Program in Engineering and Management of Natural Resources, Center for Technology and Natural Resources, Federal University of Campina Grande, Campina Grande 58429-140, Brazil; 2 Department of Chemical Engineering, Federal University of Rio Grande do Norte, Natal 59078-970, Brazil; 3 National Institute of the Semiarid, Campina Grande 58434-700, Brazil; 4 Post-Graduate Program in Process Engineering, Center for Science and Technology, Federal University of Campina Grande, Campina Grande 58429-140, Brazil; 5 Department of Food Engineering, Federal University of Campina Grande, Campina Grande 58429-140, Brazil

**Keywords:** *Opuntia*, probiotics, mucilage, dietary fiber, food safety

## Abstract

The growing interest in a healthy lifestyle has contributed to disseminating perspectives on more sustainable natural resource management. This review describes promising aspects of using cacti in the food industry, addressing sustainable, nutritional, and functional aspects of the plant’s production. Our study provides an overview of the potential of cacti for the food industry to encourage the sustainable cultivation of underutilized cactus species and their commercial exploitation. The commercial production of cacti has advantages over other agricultural practices by mitigating damage to ecosystems and encouraging migration to sustainable agriculture. The application of cactus ingredients in food development has been broad, whether in producing breads, jellies, gums, dyes, probiotics, and postbiotic and paraprobiotic foods. However, in the field of probiotic foods, future research should focus on technologies applied in processing and researching interactions between probiotics and raw materials to determine the functionality and bioactivity of products.

## 1. Introduction

Cactus plants, belonging to the *Cactaceae* family, have been an essential part of traditional diets in arid and semi-arid regions for centuries. With the growing interest in sustainable and health-promoting food sources, the potential for cacti as a functional food ingredient has gained significant attention. Cacti are well adapted to harsh environmental conditions, making them suitable for cultivation in areas with limited water resources. The unique combination of nutritional and functional properties found in cactus plants, such as high dietary fiber content, a rich array of phytochemicals, and low caloric value, offers remarkable opportunities for the food industry.

Cacti can be an important source of raw material for the production of functional foods because their consumption contributes to the intake of dietary fiber, minerals, phenolic compounds, ascorbic acid, and other antioxidant components that are related to hypoglycemic, antiobesogenic, and anti-inflammatory effects [1]. In addition, cacti have ecological importance in preventing and mitigating soil impoverishment, in addition to the fact that due to its peculiar acid metabolism, this plant can be a potential alternative to capture part of the increase in CO_2_, which causes the increase in the temperature of the soil planet [2]. Therefore, the appreciation of these plants is a promise of the future for a sustainable food production system.

Species of cacti, such as the *Opuntia* genus, are used as food and in folk medicine due to their chemical composition, which gives them enormous potential for application as a functional food [2]. The genus *Opuntia* is portrayed by Daniloski et al. [3] as one of the essential cactus species in commercial agriculture because it has many benefits that are related to its high fiber content, phytochemical characteristics, nutritional composition, and the presence of different free amino acids, which are found in the stem, fruits, and flowers [3]. However, cacti, in general, are exploited especially as a fodder resource and for animal feed [4,5,6].

The use of cacti in the pharmaceutical sector and the development of functional foods still needs to be explored, given the potential of this plant. Several literature reviews have demonstrated the beneficial health effects of parts of the plant and their extracts, claiming antioxidant, healing, skin-protective, hepatoprotective, anticancer, antiseptic, antihypercholesterolemic, and antiobesity properties [7,8,9]. Therefore, the genus *Opuntia* is a valuable and diverse source of biologically active compounds and nutrients that can be used to prepare functional foods [3]. To contribute to developing innovative food products, underutilized plants, such as several cactus species, have enormous potential for technological applications.

Cactus plants hold great promise for the food industry as a sustainable and nutrient-dense ingredient. The diverse range of potential applications and the health benefits of cactus consumption present ample product development and innovation opportunities. Therefore, this review provides and discusses the promising aspects of the use of cacti in the food industry, addressing sustainable aspects of plant production, nutritional and functional properties, as well as their potential for use in the preparation of prebiotic, probiotic, postbiotic, and paraprobiotic products, since probiotics and their derivatives are a group of functional foods on the rise and little explored, referring to products with live, inactive probiotic bacteria or their components formed during fermentation and/or released by the lysis of bacterial cells [10]. All this effort to introduce derivatives of cactus species in the food industry aims to show the potential and encourage the sustainable cultivation of cactus species and their commercial exploitation.

## 2. Importance of Cacti in the Semi-Arid Region

The world’s drylands cover nearly 47% of the Earth’s surface and support 39% of the world’s population [11]. Brazil has a semi-arid land area of 1,128,697 km^2^, home to approximately 27.9 million people [12]. In this region, a primarily low-income population is recorded, where agriculture is a primary means of survival.

As climate change has already caused significant changes in the planet and agriculture makes up a significant share of environmental degradation, they are leading to the deforestation of native areas, soil disturbance, changes in the hydrological cycle, and higher carbon emissions, along with reduced rainfall and intensified drought events [13]. These environmental changes certainly culminate in impacts on the South American dry forest biome. In this scenario, a mitigating alternative for the changes already observed is the expansion of the existing plants and their partial replacement by vegetation more suitable for arid and semi-arid regions, such as cacti [12].

**Figure 1:**
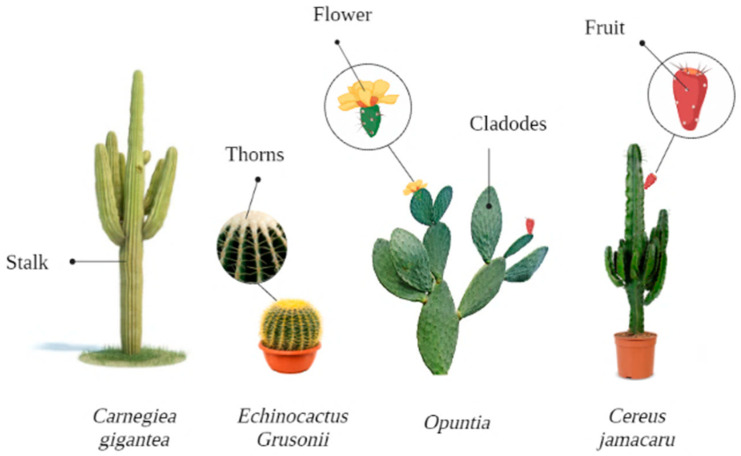
Morphological diversity of the main cactus species.

The cactus is a vital fodder resource for arid and semi-arid regions. However, in Brazil, Tunisia, South Africa, and Morocco, cactus orchards could be multipurpose, whether in human or animal food [14]. Cacti are part of a group of xerophytic plants, of the *Cactaceae* family, which have a wide variety of shapes and sizes (Figure 1), including arboreal, shrubs, globular, columnar, crawlers, tall, or dwarf, and have succulent stems—which allow them to accumulate large amounts of water in their tissues—and edible fruit [15,16,17].

**Figure 2:**
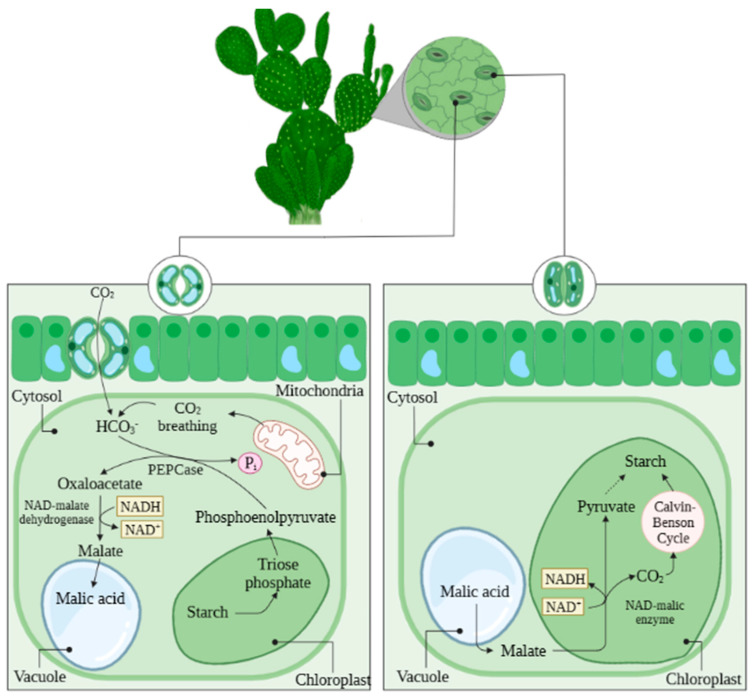
Representation of crassulacean acid metabolism (MAC) during night and day of cactus pear.

The hardiness of cacti is mainly due to their crassulacean acid metabolism, known as CAM, which favors the efficient use of water, fixing carbon dioxide (CO_2_) with phosphoenolpyruvate (PEP-carboxylase) during the night when water losses through transpiration are minimal [18] (Figure 2). This accumulation of organic acids in vacuoles overnight can reduce leaf water potential and maximize water uptake and storage, and also prevent the exchange of internal CO_2_ with the external atmosphere.

CAM plants temporarily separate carbon fixation through PEPC (phosphoenolpyruvate carboxylase) and carbon refixation through Rubisco (the first step of the Calvin–Benson cycle). When operating in CAM mode, the plant opens its stomata at night, and the diffusing CO_2_ in the leaf is fixed by PEPC, with the resulting malic acid stored in vacuoles. During the day, when the stomata are generally closed, malic acid is transported out of the vacuole, decarboxylated, and the CO_2_ is released again for final fixation by RubisCO [19]. This mechanism allows CAM plants to perform photosynthesis the following day while the stomata are closed [20]. During this process, CAM plants produce various bioactive compounds such as carboxylic acids, lignins, flavonoids, and phenols. These compounds are produced by the plant in response to environmental stresses such as water scarcity and high temperatures.

The importance of this plant is particularly evident in arid and semi-arid environments, where access to most vegetables is relatively poor, and it is mainly used as fodder in animal feed [21]. Some species are now cultivated and evaluated for fruit production [22]. These plants are an alternative source of vegetable food, nutritionally rich, and have potential in the social and economic development of vulnerable regions, where diversity and access to food are a challenge faced in the struggle to eradicate hunger.

Species of Brazilian cacti that are still little studied can be a source of food and components with technological properties for possible applications in the food industry, such as *Opuntia cochenillifera*, *Cereus jamacaru*, *Cereus hildmannianus*, *Pilosocereus gounellei*, *Tacinga inamoena*, and *Pilosocereus pachycladus* [23]. Some species considered safe for human consumption are summarized in Table 1.

Young cladodes are consumed fresh or cooked, as green vegetables in salads, soups, drinks, and sauces. They are considered important sources of nutrition in countries such as Mexico, where the consumption of these products is already widespread [21]. In this scenario, valuing cactus species from the Brazilian semi-arid region as a source of food for humans contributes to alleviating the effects of climate change and increases the diversity of highly nutritious foods, especially for vulnerable populations, in addition to generating incentives for family farming and strengthening the use of agroecological practices for the production of innovative foods.

## 3. Contemplating an Integrative and Circular Production of Cactus Species for Human Consumption Considering the United Nations SDGs

In 2015, in response to challenges related to the global biodiversity crisis and the fact that 795 million people suffer from food insecurity, world leaders committed to the Sustainable Development Goals (SDGs), which seek, among other objectives, to eradicate hunger and global poverty by 2030 [24]. However, the challenge of eliminating hunger by 2030 is even more significant for many countries, particularly in Africa, Asia, and Latin America, where, due to the economic turmoil caused by COVID-19, and in the absence of swift and effective action, more than 265 million people could become severely food-insecure [25].

Food security comprises four interrelated dimensions: production or availability of sufficient food to meet demand, good use of food, and the stability of these factors over time [26]. In addition, to ensure basic food security, it is necessary to understand the perception of different populations regarding food choices. The acceptance of foods that are not directly introduced into the food context of a given population reflects different perspectives on the same object of study that refer to historical, geographic, climatic, and cultural factors that directly influence the affective and cognitive interests of the population [1].

Cacti, as a human food, have significant economic and functional potential. In Mexico, cladodes and fruits have been used as a traditional food since the Aztec Empire in more than 200 culinary preparations; in the USA and in European and Asian countries, cladodes and fruits are considered exotic; in Brazil, cacti are considered a non-alcoholic traditional food [1]. Although social and cultural issues related to food are one of the main obstacles to developing innovative products using non-traditional food sources, valuing these products raises encouraging prospects for sustainable food production. Additionally, with the population’s growing interest in healthy foods, innovative products tend to be well accepted when presenting nutritional qualities and potential benefits to the health and well-being of consumers.

Overcoming the obstacles faced by inserting non-conventional foods into the eating habits of a population is an arduous task that requires actions to disseminate knowledge about the benefits to human nutrition and health. However, the use of cactus species in human food presents excellent possibilities for the sustainable development of agriculture, since they have advantages over other agricultural activities because of practices that mitigate, avoid, and even improve damage to the ecosystem [27]. The commercial production of cacti, through adopting sustainable agricultural practices and circular economy strategies, presents promising potential and a solution that seeks to meet the SDGs defined by the United Nations. This is only possible with the use of renewable biological resources to transform them into high-end commodities and to conserve the value of the resources for a more extended period, with the goal of zero waste generation and a reduction in greenhouse gas emissions [28]. The production of cacti for human consumption presents great potential for the use of circular economy strategies since it aims at the concept of “waste to wealth”, which gives rise to new technologies, jobs, and livelihoods, along with intrinsic benefits to the environment [28,29].

## 4. Relevant Cacti for Food Industry

Cacti are used for various purposes, including food. *Opuntia ficus-indica* is a spineless cactus that has been used for almost 500 years as a fruit crop, a defensive hedge, a support for the production of cochineal dye, as a fodder crop, and as a standing buffer feed for drought periods [30]. It is also used in erosion control and land rehabilitation, particularly in arid and semi-arid zones, and as a wildlife shelter, refuge, and feed resource [31,32]. Local populations use some species of cacti as a food source, such as the fruits and cladodes of *Cereus jamacaru* DC. In addition, the native cactus *Opuntia monacantha* has been used to obtain flour and mucilage that can be used as a hydrocolloid for various food applications. Pitaya (*Hylocereus* sp.) pulp has also been used to develop ice cream [33,34,35]. Table 2 presents the chemical compositions of the most relevant cacti for the food industry.

Cactus fruits, particularly nopal cladodes, have a higher protein content than other commonly consumed fruits. Nopal cladodes contain 1.2–2.0 g of protein per 100 g, which is higher than apples (0.3 g/100 g), oranges (1 g/100 g), and bananas (1.1 g/100 g) [36]. This makes cactus fruits a valuable source of plant-based proteins essential for maintaining the body’s cellular structure and function. However, the protein content of this food product is lower than that of staple grains such as rice and wheat. Nopal cladodes contain 1.2–2.0 g of protein per 100 g, while dragon fruit has a protein content of 0.2–1.2 g/100 g [37]. In comparison, rice contains 2.6 g/100 g of protein, and wheat has 12.6 g/100 g. However, the protein content in nopal cladodes and dragon fruit is significantly higher than in common vegetables such as lettuce (1.4 g/100 g) and cucumber (0.6 g/100 g) [37].

Dragon fruit is a notable source of carbohydrates, with a concentration ranging from 8.0 to 18.0 g/100 g. This is comparable to bananas, which contain 22.8 g/100 g of carbohydrates, and higher than apples (11.4 g/100 g) and oranges (8.5 g/100 g) [38]. This is comparable to vegetables such as potatoes, which contain 17.5 g/100 g of carbohydrates [39]. However, the carbohydrate content in dragon fruit is understandably lower than staple grains such as rice (28.7 g/100 g) and wheat (71.2 g/100 g) [40]. Despite this, its relatively high carbohydrate content positions dragon fruit as a potential energy source in meals.

Prickly pear fruit is a notable source of dietary fiber, with a content of 3.0–5.0 g/100 g, which surpasses the fiber content in apples (2.4 g/100 g), oranges (2.0 g/100 g), and bananas (2.6 g/100 g) [41]. The fiber content also surpasses the fiber content in staple vegetables such as broccoli (2.6 g/100 g) and grains such as rice (0.4 g/100 g) and wheat (1.2 g/100 g) [42].

Regarding mineral content, cactus fruits exhibit superiority over commonly consumed fruits. The calcium concentration in nopal cladodes, for example, ranges from 220 to 320 mg/100 mg, which significantly outweighs that in oranges (40 mg/100 mg), apples (6 mg/100 mg), and bananas (5 mg/100 mg) [43]. Additionally, the calcium content in nopal cladodes (220–320 mg/100 mg) significantly surpasses common vegetables such as spinach (99 mg/100 g) and grains such as rice (28 mg/100 g) [44]. This showcases the potential role of cacti as an abundant source of this vital mineral, contributing to bone health and muscle function.

The vitamin content in cactus fruits also warrants attention. Prickly pear fruit contains 10–40 mg/100 g of vitamin C, and Peruvian apple cactus fruit contains 5–20 mg/100 g [36]. These concentrations are higher than that in apples (4.6 mg/100 g) but lower than oranges (53.2 mg/100 g) and bananas (8.7 mg/100 g). These findings underscore the importance of cactus fruits as a potential source of essential vitamins.

### 4.1. Opuntia ficus-indica

*Opuntia ficus-indica*, commonly known as prickly pear or nopal cactus, is a dicotyledonous angiosperm plant that grows wild in arid and semi-arid regions of the world. It is a good source of dietary fiber, vitamins, and bioactive compounds [45]. The identified natural compounds and derivatives have been shown to have anti-inflammatory, antioxidant, hypoglycemic, antimicrobial, and neuroprotective properties [46]. The fruit of *Opuntia ficus-indica* is enriched in vitamin E at amounts up to 17.6 g/kg of α-tocopherol [46]. The cactus plant has high water-use efficiency, is drought-tolerant, and survives under erratic and low rainfall. It is highly useful in arid and semi-arid environments, particularly during prolonged dry periods [47]. In Tunisia, *Opuntia ficus-indica* constitutes a future fruit tree, mainly due to its edible fruit and vegetal mass used as food [48]. A rapid in situ propagation method of the prickly pear cactus was developed using varied portions of *Opuntia ficus-indica* cladodes harvested in spring or autumn, planted horizontally or vertically to optimize rhizogenesis and secondary cladode initiation rates. Older prickly pads were found to be an important source of nutritional components such as calcium. Mineral content revealed that K was highest in the fruit and Ca was highest in the stem [49].

*Opuntia ficus-indica* contains bioactive compounds such as pigments (carotenoids, betalains, and betacyanins), vitamins, flavonoids (isorhamnetin, kaempferol, and quercetin), phenolic compounds, and minerals [50,51]. These compounds contribute to the antioxidant activity of *Opuntia ficus-indica* by scavenging free radicals and reducing oxidative stress [52]. The peels of *Opuntia ficus-indica* have been found to have higher yields of total phenolic compounds and flavonoids compared to the juicy pulp [53]. The antioxidant activity of *Opuntia ficus-indica* has been evaluated through the reduction potential and the inhibitory effect of DPPH in peel extract. The total phenolic compound content in *Opuntia* spp. is quite variable and is affected by the maturity stage, harvest season, environmental conditions, postharvest treatment, and species [50]. Together, these vitamins and minerals contribute to the nutritional value of *Opuntia ficus-indica*.

### 4.2. Cereus peruvianus

This cactus is known as Peruvian apple cactus, and its fruit is often eaten fresh or used in jams, jellies, and beverages. *Cereus peruvianus* is a cactus plant that has been studied for its composition is some studies. The carbohydrate contents of polysaccharides extracted from *Cereus peruvianus* stems and calli tissues were estimated to be 58.08% and 35.21%, respectively. The glycosyl composition of the stems and callus polysaccharides consisted mostly of galactose, arabinose, and rhamnose [54]. The mucilage of *Cereus peruvianus* has been found to have some similarity to both pectic polysaccharides and gum exudates, and may provide an alternative source of industrial raw materials [55]. *Cereus peruvianus* is a cactus plant that has been used for ornamental purposes. Somaclones with typical and atypical shoots were regenerated from a callus culture in the same culture medium and in equal culture conditions. The low molecular divergence between typical and atypical morphologies of somaclones is promising for the use of atypical somaclones as a source of chemical compounds of commercial and industrial interest [56]. Two-dimensional electrophoresis of *Cereus peruvianus* callus tissues grown in culture media containing two different 2,4-dichlorophenoxyacetic acid (2,4-D) and kinetin combinations was used to identify minor differences in the polypeptide composition of these cell clones [57]. Overall, more research is needed to fully characterize the composition of *Cereus peruvianus*.

### 4.3. Hylocereus undatus

*Hylocereus undatus*, also known as dragon fruit, is a fruit that is rich in several phytochemicals and is supposed to be very nutritious. It contains high amounts of antioxidants, minerals, and a balanced content of nutrients. High amounts of dietary fiber and carotenoids make it beneficial for chronic heart disorders, cancer, and diabetes. The fruit pulp can be dehydrated into a powder form while keeping the nutritive value intact. This increases the shelf life and makes it easier to distribute and transport the fruit. The seeds of *Hylocereus undatus* contain methyl hexadecanoate (C16:0), methyl trans-9-octadecenoate (C18:19 trans), and methyl cis-11-octadecenoate (C18:111 cis) of 15 kinds of fatty acids. The vitamin C content in *Hylocereus undatus* ranges from 10.00 ± 1.14 mg to 217.90 ± 3.01 mg/100 g of edible portion of fruits. Copper and zinc are found in high amounts in Chapa kola (*Musa* spp.), while iron and manganese are found in high amounts in Bangla kola (*Musa* spp.). The foliage and peels of *Hylocereus undatus* were extracted using two different solvents, namely chloroform and methanol, through the Folin–Ciocalteu method and the Diphenyl-1-Ipicrylhydrazyl (DPPH) free radical scavenging assay for total phenolic content (TPC) and antioxidant activity, respectively. Results revealed that the peels gave higher TPC in both methanol (48.15 mg GAE/100 g extract) and chloroform (18.89 mg GAE/100 g extract) extractions than foliage (30.3 mg GAE/100 g extract and 5.92 mg GAE/100 g extract, respectively). However, when a comparison was made between foliage and peels in terms of their scavenging effects in the DPPH assay, the peels showed higher antioxidant activity than foliage.

In the work by Som et al. [58], the antioxidant activity of *Hylocereus undatus* foliage was compared to its peel. The study found that the peels gave higher total phenolic content (TPC) in both methanol and chloroform extractions than foliage. However, when a comparison was made between foliage and peels in terms of their scavenging effects in the DPPH assay, the peels showed higher antioxidant activity than foliage. Therefore, the antioxidant activity of *Hylocereus undatus* peel is higher than its foliage.

### 4.4. Potential Toxins and Allergens from Cacti

It is important to consider safety and potential risks when consuming any new food product, including cacti and their derivatives. Some cacti can contain compounds that may have toxic effects when ingested in large amounts. For example, certain species of cacti contain substances such as mescaline, a psychoactive compound, or oxalic acid, which can lead to kidney stones if consumed excessively. However, the types of cacti commonly used for food, such as the prickly pear cactus (*Opuntia ficus-indica*), are generally considered safe to eat [59]. Some individuals may have allergies to cactus or cactus products. Symptoms of an allergic reaction can range from mild (e.g., a rash or itchy skin) to severe (such as difficulty breathing) [60].

Additionally, glochids are tiny, hair-like spines found on some cacti, including the prickly pear. They can easily detach and get stuck in the skin, causing irritation. Therefore, it is crucial to handle these cacti carefully and ensure they are thoroughly cleaned and prepared before consumption [61].

As with any food product, the quality of cactus-based products can vary depending on factors such as the growing conditions of the cactus, the harvesting and processing methods used, and the quality control measures in place. Consumers should look for products that meet food safety standards and are compliant with relevant regulations [62].

## 5. Functional Ingredients Derived from Cactus Species for the Food Industry

Cacti are still little explored, but they have great potential for developing innovative food products due to their bioactive components, polysaccharides, and other constituents of interest in areas related to the study and development of food, pharmacology, and biotechnology. Due to their advantages and benefits, including their ability to adapt well to arid and semi-arid environments and their nutritional values, cactus cultivars are considered a functional food for the future and a potential crop for expanding the food base, thus ensuring food safety [21]. Table 3 summarizes cacti components and their applications in the food industry, along with health benefits.

### 5.1. Dietary Fiber

Dietary fiber is a fundamental component of a balanced diet, known for its multiple health benefits, including its role in promoting gut health, supporting weight management, and maintaining healthy blood sugar levels. Cacti, particularly the *Opuntia* spp. (nopal cactus), are abundant in dietary fiber, making them excellent candidates for the development of fiber-rich food products [63]. Table 4 summarizes some works regarding the health benefits of prebiotic food products formulated with cacti products.

The dietary fiber in cacti is composed of both soluble and insoluble forms [69]. Soluble fiber forms a gel-like substance in the gut, aiding digestion by slowing down the emptying of the stomach, which can contribute to feelings of satiety and help regulate blood glucose levels. On the other hand, insoluble fiber adds bulk to the diet, helping prevent constipation and promoting regular bowel movements.

In the food industry, the high fiber content of cacti can serve multiple purposes. First, it can be used as a natural thickening agent in various food products such as sauces, soups, and bakery items, improving their texture and mouthfeel. Hussain et al. [70] reported decreased bread hardness after 24 h when using cactus gum from *Opuntia ficus-indica* compared to acacia seyal gum, which is another dietary fiber. Second, cactus fiber can enhance the nutritional profile of food products, meeting the demand of health-conscious consumers seeking out high-fiber diets. Uebelhack et al. [71] reported that cactus fiber significantly promoted fecal fat excretion in healthy adults, hypothesizing that it reduces body weight by binding to dietary fat. Lastly, some studies suggest that cactus fiber might reduce calorie intake by inducing satiety. This feature can be leveraged in the development of weight-management food products [72].

#### 5.1.1. Mucilage

Another component present in cacti with great potential for exploitation is mucilage. Mucilage, or natural hydrocolloids, are complex carbohydrates capable of absorbing water that can act as thickening agents and stabilizers, with applications in several areas, such as base materials for edible coatings, bioplastics, encapsulating agents, and in the formulation of gluten-free foods [23]. Furthermore, the presence of monosaccharides such as xylose indicates a prebiotic potential of the mucilage of some cactus species. Therefore, it is essential to exhaustively describe all the compounds of these by-products and their related biological properties to promote their application in the food, pharmaceutical, and cosmetic industries, promoting a circular economy [73].

#### 5.1.2. Pectin

Pectin is a complex carbohydrate, or polysaccharide, found in the cell walls of plants, where it plays a crucial role in cell growth and plant structure. In the context of cacti, the prickly pear (*Opuntia* spp.) is known to contain significant amounts of pectin, particularly in its fruit and cladodes (the plant’s flattened branches) [74]. Pectin is a highly valued ingredient in the food industry due to its gelling, thickening, and stabilizing properties. It is widely used in the production of jellies, jams, marmalades, and certain types of candy due to its ability to form a gel in the presence of sugar and acid. It can also be used as a fat substitute in baked goods to stabilize acidic protein drinks such as yogurt [75].

Cactus-derived pectin has been shown to have good gelling characteristics comparable to commercial pectin, as proven by Goycoolea and Cárdenas [76]. Costa et al. [77], using both acid and enzymatic extraction methods, yielded high-quality pectin from the peels of *H. undatus* and *H. costaricencis* with acid extraction, producing pectin with higher degrees of methyl-esterification and galacturonic acid content, while enzymatic extraction yielded pectin with higher molar mass. It also exhibits excellent water-holding and fat-binding capacities, expanding its potential uses in the food industry. For instance, it can improve texture, reduce syneresis (liquid separation), and increase the shelf life of various food products [78]. Mohamed et al. [79] also reported the cost-effective usage of pectin-rich cactus pear peel as a bio-based adsorbent.

In addition to its functional properties, cactus pectin also offers potential health benefits. Pectin is a type of dietary fiber that can promote gut health, aid in controlling blood sugar levels, and help lower cholesterol levels. Moreover, research has suggested that pectin may have prebiotic properties, promoting the growth of beneficial gut bacteria [80]. In conclusion, extracting pectin from cacti, particularly from species such as *Opuntia* that are abundant and easy to cultivate, provides an excellent opportunity for the sustainable production of this valuable food ingredient. However, more research is needed to optimize extraction methods and better understand cactus-derived pectin’s unique properties.

### 5.2. Phytochemicals

Phytochemicals are bioactive compounds found in plant foods associated with various health benefits. Table 5 presents a summary of works that report the health benefits of food products formulated with cacti products. Cactus species, particularly *Opuntia* spp. and *Hylocereus* spp., are rich in several phytochemicals, including polyphenols, flavonoids, and betalains. These compounds have attracted considerable interest due to their potent antioxidant, anti-inflammatory, and potential anticancer properties.

#### 5.2.1. Polyphenols

In addition to its use as food, species of the genus *Opuntia* have a vast potential for non-food use, exploring their biofunctional, medicinal, nutraceutical, and cosmetic properties [27]. *Opuntia* species have been used for centuries as food resources and in folk medicine for treating chronic diseases such as obesity, cardiovascular and inflammatory diseases, diabetes, and gastric ulcers [50]. They are also a source of food coloring agents and present exciting possibilities for producing bioethanol and biogas from cladodes [27].

**Figure 3:**
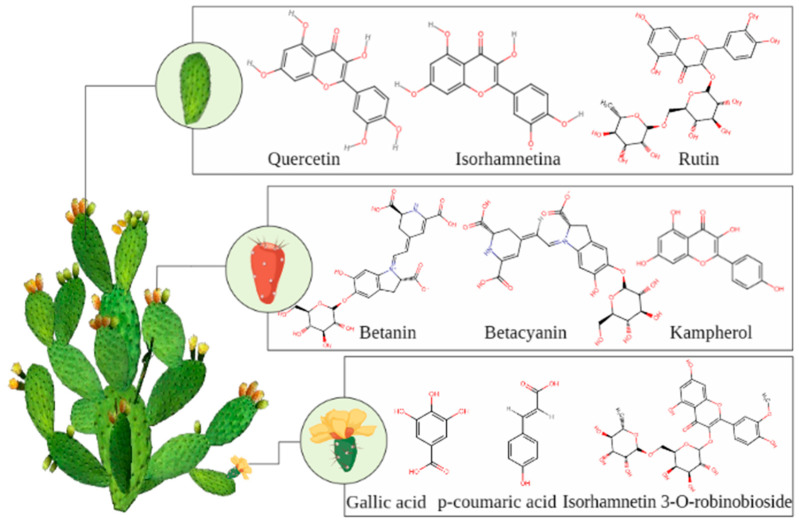
Main compounds present in cacti. Chemical structures were drawn using PubChem (https://pubchem-ncbi-nlm-nih.ez292.periodicos.capes.gov.br, accessed on 21 February 2023).

The relationship between the potential of the fruits and cladodes of cacti and health benefits is due to the presence of secondary metabolites such as phenolic compounds, carotenoids, and betalains [91] (Figure 3). Of the cactus fruits, prickly pear (*Opuntia ficus-indica*) is one of the most studied fruits of the *Cactaceae* family, especially the biological activities of betalains and flavonoids, which have shown high antioxidant potential and antihyperglycemic and anti-inflammatory activity [92]. However, other species have also been explored in this sense with the aim of enabling the sustainable production of bioactive compounds, such as safe belatains [91] and the extraction of other polyphenolic compounds [93].

The fruits contain phenolic alkaloids, such as betacyanins and betaxanthin, and glycosylated flavonoids that exhibit various pharmacological activities. In addition, red and yellow betalains, being water soluble, are used as a natural food coloring [94]. Therefore, varieties of *Opuntia* can be used as a source of natural antioxidants suitable for the development of different functional food products, because in addition to the components already mentioned here, varieties of *Opuntia* are rich in other antioxidants present in all parts of the plant, such as lignans, sterols, esters, saponins, and alkaloids, all of which have shown potential protective effects against various chronic diseases [3].

In cactus cladodes, the components found are known to combat oxidative stress. In addition, they are a source of soluble and insoluble fibers that are not absorbed by the human digestive system, and therefore are used to regulate weight, increase the intake of fiber, control diabetes mellitus, and as a natural source of potassium [22]. In addition to fibers, the composition of cactus cladodes includes pectin, mucilage, lignin, cellulose, and hemicellulose [95]. The composition of cactus cladodes varies under many factors, such as age, season, variety, soil type, growing conditions, and climate [96]. This is the case for bioactive compounds, such as polyphenols and carotenoids with relevant antioxidant activity [97]. Flavonoids are the primary polyphenols in cactus cladodes, representing about 85% of the total polyphenols, consisting of flavanols such as isorhamnetin present in greater abundance, followed by a smaller amount of quercetin and kaempferol [98].

#### 5.2.2. Flavonoids

Flavonoids are a group of polyphenolic compounds produced in plants as secondary metabolites and have favorable biochemical effects on multiple diseases as well as other bioactivities such as anti-inflammatory and antiaging [99]. In their chemical structure, flavonoids have three C6-C3-C6 rings, which are generally classified into seven subclasses according to their structural differences: flavones, isoflavones, anthocyanidins, flavanones, flavanols, and chalcones [99].

In cactus cladodes, the most abundant compound is isorhamnetin, which can also be found in flowers. Isorhamnetin has many pharmacological actions, including antioxidant, anti-inflammatory, antimicrobial, anticancer, hepatoprotective, reproductive-system-protective, anti-obesity, and antidiabetic [100]. The pharmacological effects of isorhamnetin are related to its regulation of NF-kB, PI3K/AKT, MAPK, and other signaling pathways and their downstream factors [101]. This component in the plant *Opuntia* species is a subject of great interest among researchers from different areas, especially food pharmacology, due to its beneficial actions for humans, which drives the exploration of this and other components for functional food and drugs to aid in treating diseases.

### 5.3. Oil from Cactus Sources

Certain cactus species, such as *Opuntia ficus-indica*, are known for their seed oil, often called prickly pear seed oil. The oil is obtained from the tiny, hard seeds in the cactus fruit. The oil yield from these seeds is usually low, but the resultant oil is highly valued due to its unique properties and potential benefits [102]. Table 6 summarizes the fatty acid profile of prickly pear seed oil and some properties of its oil.

Prickly pear seed oil is rich in linoleic acid, an essential fatty acid, and tocopherols, including vitamin E [103]. These compounds give the oil strong antioxidant properties. The cactus seed oil is also characterized by its high sterol content, including β-sitosterol, associated with cholesterol-lowering effects [102]. Cactus seed oil can be used as a cooking oil in the food industry due to its relatively high smoke point [104]. However, it is more commonly used as a functional ingredient in food formulations to enhance nutritional value and deliver potential health benefits. For example, it can be used in salad dressings, dips, and spreads, or as a carrier oil for other flavors and bioactive ingredients.

Furthermore, the oil has a pleasant taste and aroma, which can enhance the sensory appeal of food products. However, the extraction of oil from cactus seeds is a labor-intensive process which, combined with the low yield, contributes to the high cost of this oil. Therefore, research is ongoing to optimize the oil extraction process and better understand this oil’s full potential in the food industry and other sectors [105].

**Table 6:** Fatty acid profile, physical and chemical properties, and sensory properties of cactus seed oil.

Parameter	Typical Value
Fatty Acid Profile [106]	
Myristic acid 14:0	0.10
Palmitic acid 16:0	12.29
Palmitoleic acid 16:1 (9Z)	0.75
7Z, 10Z-Hexadecadienoic acid, 16:2 (6Z, 9Z)	0.05
Stearic acid 18:0	3.92
Oleic acid 18:1 (9Z)	17.61
Vaccenic acid 18:1 (11E)	6.29
13-Octadecanoic acid 18:1 (13E)	0.17
Linoleic acid 18:2 (9Z, 12Z)	57.98
Linolenic acid 18:3 (9Z, 12Z, 15Z)	0.21
Arachidic acid 20:0	0.33
Gondoic acid 20:1 (11Z)	0.10
Methyl-9-eicosenoate	0.22
Physical Properties [107]	
Density (g/cm^3^ at 20 °C)	~0.92
Refractive index (at 20 °C)	~1.47
Smoke point (°C)	~200
Chemical Properties	
Iodine value (g I_2_/100g)	~90–100
Acid value (mg KOH/g)	~1
Peroxide value (meq O_2_/kg)	~10
Sensory Properties	
Color	Yellow to greenish
Aroma/flavor	Light, nutty

## 6. Future Trends for Technological Application of Cacti-Derived Ingredients

Although the use of cactus-derived ingredients presents technological and marketing challenges, they present some nutritional, functional, and technological advantages when compared to other ingredients, such as wheat, artificial colors, and hydrocolloids [3,106]. Additionally, with population growth, natural resources face continual depletion. As such, there is renewed interest in sustainably mobilizing sources of functional ingredients, thereby preventing a loss of bioactivity and promoting the sustainable use of the Earth’s ecosystem [73]. Therefore, these characteristics give this food matrix a high potential for developing products in the food industry.

### 6.1. Bakery

In this sense, incorporating alternative ingredients into food products represents a promising strategy for developing nutritious, sustainable, agroecological products [73]. Machado et al. [107] stated that xique-xique biscuits can be considered an innovative product for the functional food industry, mainly due to their bioactive and nutritional characteristics, being excellent sources of fiber and minerals, which are essential nutrients for the general population. In addition to the richness of active and functional biomolecules, the presence of fibers and polyphenols gives biscuits technological properties such as kneading ability, flavor retention, and antioxidant capacity [108].

According to Msaddak et al. [109], *Opuntia ficus-indica* cladodes can be considered a potential health-promoting functional ingredient in bakery products. In this study, the authors observed that replacing wheat flour with cladode powder at 5% improved the total phenolics content and the bread’s antioxidant potential without negatively affecting its sensory acceptability. Dick et al. [97] reported that crackers with 2% cactus mucilage and 5% clacode flour received the highest acceptance in sensory evaluations, suggesting their potential as appealing and nutritious alternatives in gluten-free cracker formulation. Ali et al. [110] reported the use of roasted prickly pear seed flour in the nutritional and sensory characteristics of bread and found significant increases in the dietary fibers, fat, and ash contents, phenolic concentration, and antioxidant activity while the sensory properties of the bread were not affected at levels up to 6% supplementation. However, above 6%, the bread became unacceptable.

As mentioned earlier, the studies suggest that cacti, particularly the *Opuntia* species, hold promise as functional ingredients in bakery and pastry products. Their high dietary fiber and antioxidant content can enhance the nutritional profile of these products. However, the impact on sensory characteristics must be carefully considered and optimized to ensure consumer acceptance.

### 6.2. Food Dyes

The food industry is increasingly interested in using natural food dyes to replace harmful synthetic dyes [111]. Thus, some studies using cacti-derived ingredients as natural pigments are available in the literature. Pigments from the pulp of *Hylocereus polyrhizus* in encapsulated form were obtained by [112], showing themselves as potential natural dyes with characteristics suitable for application in food. Carmona et al. [113] used microparticles of orange-yellow cactus pear pulp (*Opuntia ficus-indica*) as a natural yellow colorant in yogurts. Ruiz-Gutiérrez et al. [114] used encapsulated powder from red cactus pear (*Opuntia ficus-indica*) to pigment extruded cereal, showing in this study that encapsulated powder from red palm fruit can be a natural alternative to synthetic dyes. It can be used to develop functional foods with possible health benefits. Lugo-Zarate et al. [115] stated that the addition of purple cactus pear powder can provide an intense color as a natural additive for dairy products such as yogurt, which can be an alternative to synthetic additives since they play an essential role in product quality—and consumer acceptance. In addition, purple palm juice powder added to yoghurt also provides antioxidant content and high bioaccessibility, with slight changes in its physicochemical characteristics.

According to Nunes et al. [116], the technological applications and the economic potential of these species can also be extended to their consumption in the form of gum candies, sweets, and juices. In the current literature, Oliveira et al. [117] elaborated on a new mixed drink formulated with juice from the cladode xique-xique (*Pilosocereus gonellei*). The authors reported that incorporating cladode juice from xique-xique into these mixed drinks potentiated its bioactive properties, mainly of antioxidant compounds, allowing the development of a new product with potential functional properties for the beverage industry. Moussa-Ayoub et al. [118], when developing *Opuntia dillenii* cactus juice, exposed that the juices produced had high bioactive potential and favorable characteristics beneficial to human health.

Gummies added with encapsulated betalains from *Opuntia ficus-indica* were developed by Otálora et al. [119]. These candies showed gelling and morphological properties suitable for use in the confectionery industry. Furthermore, the food system sample stored at 4 °C for 30 days did not significantly change its color. Moussa-Ayoub et al. [120] evaluated the use of the whole yellow-orange fruit of the cactus pear *Opuntia ficus-indica* as an ingredient in rice- and corn-based snacks. This study showed that the unique profile of flavanols from the fruits of the cactus *Opuntia ficus-indica* could serve as a biochemical marker for evaluating the authenticity of products derived from whole palm fruits or fruit peel. Another possible application was reported in the study by Tabarestani et al. [121], in which they used the pulp of *Opuntia* as a clean label ingredient in developing a meat-free burger. The incorporation of this ingredient as a great source of phenolic components considerably influenced the cooking yield, moisture retention, juiciness, and oxidative stability of meatless burgers; in addition, the use of this ingredient could provide a sustainable burger that is beneficial to public health, the environment, and animal welfare.

### 6.3. Probiotic

Regarding probiotic products, Panda et al. [122] showed that adding *Limosilactobacillus fermentum* to prickly pear juice through fermentation provides a new functional product with various antioxidants and health-beneficial phytochemicals. Cactus pear juice fermented by *Lactiplantibacillus plantarum* subsp. *plantarum*, when administered to obese mice, caused a significant decrease in body weight gain and alleviated insulin resistance, showing the potential of the functional drink for the prevention of obesity and related pathologies [123]. Despite these reports, the development of probiotic products with cacti is scarce, and the study of the addition of postbiotics and paraprobiotics in the formulation of new food ingredients from this raw material is non-existent in the literature; therefore, the topic deserves more studies that can contribute to the scientific and technological development field of functional foods.

### 6.4. Functional Beverages

Functional beverages represent a rapidly expanding segment of the food and beverage industry. These drinks are not just consumed for hydration but also for their nutritional and health-promoting properties. Given the notable nutritional and health benefits of cacti, particularly species such as *Opuntia* spp. and *Hylocereus* spp., they are increasingly being considered for use in functional beverage development. According to the work by Stintzing et al. [124], cactus pear fruit, particularly from *Opuntia* species, boasts significant nutritional value, including high levels of amino acids, proline, and taurine, making the cactus pear a versatile ingredient in functional food preparations, notably for dairy products.

Alcántara-Zavala and Figueroa-Cárdenas [125] reported that the use of ohmic heating technology effectively prolonged the shelf life of red cactus pear pulque, better retained its quality attributes, and preserved beneficial microbial content, suggesting its potential for wider use in cactus-based beverage processing. El-Sayed and Ramadan [126] reported that the fermentation of rice milk beverages fortified with cactus pear and physalis pulps significantly improved product quality, increasing antioxidant properties and maintaining probiotic viability over 12 days of storage at 5 °C, with the beverage containing 20% cactus pear pulp showing the highest overall acceptability. Hou et al. [127] formulated a compound fruit and vegetable beverage from *Opuntia Milpa Alta* cactus, cucumber, and kiwifruit, with optimal blanching and clarification conditions determined for each ingredient. The resulting beverage, comprising 10% cactus juice, 2.5% cucumber juice, 7.5% kiwifruit juice, 6.5% sugar, and 0.08% citric acid, had a clear, stable character with a refreshing taste, unique flavor, and notable nutritional value, suggesting potential health benefits.

Tsegay and Gebremedhin [128] explored the production of cactus pear and Lantana camara fruit juice blend wine, revealing enhanced total phenol, color, and sensory value, while maintaining acceptable acidity and methanol and sulfite contents within commercial wine standards. The findings suggest that this blend could be a viable option for creating health-enhancing functional beverages, potentially addressing postharvest losses of both fruits and offering an alternative beverage option. Ayed and Hamdi [129] showcased the successful fermentation of cactus pear juice into kombucha, improving the nutritional properties. The fermentation process led to a reduced pH, increased total phenol content, enhanced antioxidant capacity, and displayed antimicrobial activity against various bacteria, attributed to its acetic acid content. After 6 days of fermentation, the cactus pear kombucha had high acceptability due to retained taste qualities, though some found it less palatable after extended fermentation due to increased acidity.

Given these trends, the future for cacti in the functional beverage industry appears promising. As more research is conducted into the health benefits of cacti, and as consumer demand for healthier and more sustainable food options continues to grow, it is expected that we will see a broader range of cactus-based beverages on the market in the coming years.

## 7. Conclusions

Amid food-related health and wellness trends, our work sheds light on the potential of bioactive components from underutilized plants, such as cacti, for exploration and application in food and pharmaceutical products. This aligns with the growing trend of developing products that are beneficial to health, considering the use of cacti for human food; the value of cacti species in the market and for economically underdeveloped regions; and their contribution to the sustainable development of the planet in view of their adaptability in arid and semi-arid regions and in mitigating the effects of climate change in the world. We have seen that phenolic compounds, dietary fibers, mucilage, and other components extracted from cacti have started to be explored by the industry, while probiotic foods are one of the most prosperous areas, with postbiotics and paraprobiotics as a future promise for the delivery of probiotic-related benefits through food. However, more needs to be explored about ways to add probiotics and their components to cactus derivatives for the development of foods and functional food inputs. To expand and diversify functional foods prepared with cacti, we propose new research on the application of cactus ingredients in developing probiotic, postbiotic, and paraprobiotic foods. Future research should focus on technologies applied in processing and on researching the interactions between components and cacti and probiotics, phenolic compounds, and other components to determine the functionality and bioactivity of the products.

## Figures and Tables

**Table 1:** Species of cacti and their traditional use in human nutrition.

Species	Edible Part	Traditional Use
*Opuntia fícus-indica* *Opuntia cochenillifera*	Cladodes	As a vegetable in salads, jelly, flour, or extracts
	Fruits	Consumption of fresh pulp, jelly, and beverage
	Flowers	Extracts used in folk medicine
*Cereus peruvianus* *Cereus jamacaru* *Cereus hilmanianus*	Cladodes	Extracts used in folk medicine
	Fruits and seeds	Consumed fresh or in culinary preparations
*Pilosocereus gounellei* *Pilosocereus pachycladus*	Cladodes	As a vegetable in salads, jelly, flour, or extracts
	Fruits	Consumed fresh or in culinary preparations
*Hylocereus undatus*	Fruits	Consumed fresh or in culinary preparations
	Flowers	Extracts used in folk medicine
*Selenicereus grandifloras*	Fruits	Consumed fresh or in culinary preparations
*Stenocereus thurberi*		
*Tacinga inamoena*		

**Table 2:** Chemical composition of the most relevant cacti for food industry.

Cacti Specie	*Opuntia ficus-indica*		*Cereus peruvianus*	*Hylocereus* spp.
Nutrient	Nopal Cladodes	Prickly Pear Fruit	Peruvian Apple Cactus Fruit	Dragon Fruit
Moisture (g/100 g)	90–94	88–92	88–92	80–90
Protein (g/100 g)	1.2–2.0	0.5–1.1	0.6–0.9	0.2–1.2
Fat (g/100 g)	0.1–0.5	0.3–0.6	0.1–0.3	0.1–0.7
Carbohydrates (g/100 g)	3.0–4.5	7.0–12.0	6.0–10.0	8.0–18.0
Dietary Fiber (g/100 g)	2.2–4.0	3.0–5.0	1.0–2.0	1.0–3.0
Ash (g/100 g)	1.1–1.6	0.8–1.2	0.4–0.7	0.4–0.8
Minerals				
Calcium (mg/100 mg)	220–320	30–55	20–30	6–15
Magnesium (mg/100 mg)	52–85	15–30	10–20	10–20
Potassium (mg/100 mg)	300–430	220–330	200–300	200–400
Sodium (mg/100 mg)	21–45	3–15	1–10	0–50
Iron (mg/100 mg)	0.5–2.0	0.3–1.5	0.2–0.5	0.3–0.7
Vitamins				
Vitamin A µg/100 g	50–100	20–50	Not found in the literature	10–40
Vitamin C mg/100 g	9–25	10–40	5–20	2–25
Vitamin E mg/100 g	0.5–1.5	0.3–1.0	Not found in the literature	Not found in the literature
Phytochemicals				
Polyphenols mg/100 g	50–200	30–100	Not found in the literature	10–30
Flavonoids mg/100 g	10–50	10–30	Not found in the literature	Not found in the literature
Betalains mg/100 g	Not significant	10–50 (peel)	Not found in the literature	10–50 (peel)

**Table 3:** Summary of cacti components and their applications in the food industry.

Cacti Component	Health Benefits	Applications in the Food Industry	References
Dietary Fiber	Promotes gut health, supports weight management, and maintains healthy blood sugar levels.	Used as a natural thickening agent in sauces, soups, and bakery items. Enhances the nutritional profile of food products. Reduces calorie intake by inducing satiety.	[63,64,65,66,67]
Mucilage	Potential prebiotic benefits due to the presence of monosaccharides such as xylose.	Used as a thickening agent and stabilizer. Applications include the development of edible coatings, bioplastics, encapsulating agents, and gluten-free foods.	[23,68]
Pectin	Promotes gut health, aids in controlling blood sugar levels, and helps lower cholesterol levels. Potential prebiotic properties.	Used for its gelling, thickening, and stabilizing properties in the production of jellies, jams, marmalades, and certain types of candy. Acts as a fat substitute in baked goods to stabilize acidic protein drinks such as yogurt.	[69,70,71,72,73,74,75]
Polyphenols	Potent antioxidant, anti-inflammatory, anticancer properties, antihyperglycemic activity, and potential benefits in obesity, cardiovascular and inflammatory diseases, diabetes, and gastric ulcers.	Used as food resources and in folk medicine, potential use in nutraceutical and cosmetic industries. Serves as a source of food coloring agents and bioethanol and biogas production. Contains beneficial compounds such as lignans, sterols, esters, saponins, and alkaloids.	[3,22,27,50,76,77,78,79,80,81,82,83]
Flavonoids	Anti-inflammatory, antiaging, antioxidant, antimicrobial, anticancer, hepatoprotective, reproductive system protective, antiobesity, and antidiabetic effects.	Significant component in the formulation of functional food and drugs. Known to regulate signaling pathways (e.g., NF-kB, PI3K/AKT, MAPK).	[84,85,86]
*Opuntia ficus-indica* (Prickly pear seed oil)	Rich in linoleic acid and tocopherols, including vitamin E. High sterol content, particularly β-sitosterol. Strong antioxidant properties. Relatively high smoke point. Pleasant taste and aroma.	Used as cooking oil; a functional ingredient in food formulations such as salad dressings, dips, and spreads; and a carrier oil for other flavors and bioactive ingredients. Additionally, valuable in the cosmetic industry.	[87,88,89,90]

**Table 4:** Studies on the prebiotic potential of cacti.

Species	Probiotic Strain	Results	Reference
*Opuntia fícus-indica* and *Opuntia joconostle*	*Lacticaseibacillus rhamnosus* GG (ATCC 53103), *Lactobacillus acidophilus* (DSM 13241), and *Bifidobacterium longum* subsp. *Infantis* (ATCC 15697)	All strains were able to use mucilage as substrate, although with lower maximum growth than glucose.	[64]
*Opuntia ficus-indica*	Colon microbiota obtained from a fecal sample from a healthy adult volunteer	The mucilage increased *Lactobacillus* growth by up to 23.8% and produced a slight decrease in enterococci, enterobacteria, staphylococci, and clostridia.	[65]
*Opuntia fícus-indica* and *Opuntia joconostle*	*Lacticaseibacillus rhamnosus* GG (ATCC 53103), *Lactobacillus acidophilus* (DSM 13241), *Bifidobacterium longum* ssp. *Infantis* (ATCC 15697), and *Bifidobacterium animalis* ssp. *Lactis* Bb-12 (DSM 15954)	Mucilage fermentability by selected probiotics was relatively low, 11–27% compared to glucose, and decreased with increasing levels of galacturonic acids in the molecules. Therefore, its fermentability by probiotic species can be attributed more to its structural characteristics and monosaccharide composition than to its dimensions.	[66]
*Pilosocereus gounellei*	*Lactobacillus acidophilus* LA-05, *Lacticaseibacillus casei* L-26, and *Lacticaseibacillus paracasei* L-10	Juice from *Pilosocereus gounellei* cladodes showed positive scores for prebiotic activity in all probiotics examined, indicating a selective stimulatory effect on these microorganisms to the detriment of enteric pathogens.	[67]
*Opuntia streptacantha*	*Lactobacillus acidphilus*, *Lacticaseibacillus casei*, and *Bifidobacterium animalis* subsp. *lactis*	The mucilage fractions stimulated the in vitro growth of commercial probiotics, with behavior similar to that obtained with commercial inulin.	[68]

**Table 5:** Evidence of potential beneficial health effects from the use of cactus derivatives.

Material	Study	Results	References
Polysaccharides extracted from *Tibet Opuntia ficus-indica* (Linn.) Mill.	Cyclophosphamate-induced immunocompromised mice	Ingestion of polysaccharides significantly regulated the relative abundance of *Lactobacillus*, *Bacterioides*, and *Akkermansia*, and the new dominant intestinal bacterial species were Deferribacteres, Actinomycetes, Firmicutes, Tenericutes, Actinomycetes, and Pasteurella. Polysaccharides can effectively increase the metabolic level of lysine synthesis and decomposition, regulate the level of gene expression after immune disorders, and improve the overall health of immunodeficient mice.	[81]
Cooked *Opuntia ficus-indica*	Sample of 36 female volunteers, with an obesity group consisting of 25 women and another group consisting of 11 women of normal weight.	It was reported that intervention with *Opuntia Ficus-indica* in the diet of women induced changes in the intestinal microbiota in both groups of women, associated with biochemical and anthropometric parameters.	[82]
Dried cladodes of *Opuntia ficus-indica*	Obese rats fed a high-fat, high-sucrose diet	The addition of *Opuntia ficus*-*indica* cladodes to the diet of rats can ameliorate specific biochemical parameters of obesity, such as total cholesterol, GIP, APP, leptin, and peptides, modifying the intestinal microbiota. Therefore, dried cladodes of *Opuntia ficus*-*indica* have the ability to modify the intestinal microbiota and reduce metabolic endotoxemia and other biochemical abnormalities related to obesity, supporting their use as a functional food and prebiotic.	[83]
Red pitaya betacyanins	Male mice fed low-fat diet, high-fat diet, and high-fat diet plus *Hylocereus polyrhizus*	The addition of red pitaya betacyanins to the diet protects against diet-induced obesity and related metabolic diseases, is associated with improving the inflammatory state, modulating the intestinal microbiota, decreasing the proportion of *Firmicutes* and *Bacteroidetes*, and increasing the relative abundance of *Akkermansia* at the genus level.	[84]
*Opuntia ficus-indica* extracts	Antioxidant activity, in vitro antitumor activity, and antimicrobial activity	Antimicrobial activity against pathogenic viruses and bacteria was observed in extracts of *Opuntia ficus-indica*. Selective cytotoxicity towards cancer cells.	[85]
*Butia* and *Opuntia* fruit hydroethanolic extracts	Antioxidant capacity in vitro and in vivo	The hydroethanolic extracts of *Butia* and *Opuntia* fruits showed antioxidant effects in vitro and in vivo, as well as no toxic effect in vivo.	[86]
*Opuntia robusta* and *Opuntia streptacantha* fruit extracts	In vitro and in vivo study with mice	Therapeutic treatments with extracts of *Opuntia* reduced biochemical, molecular, and histological markers of liver injury (in vivo) and hepatocytes (in vitro). *Opuntia* extracts reduced the increased APAP expression of the stress-related gene Gadd45b, and exerted diverse effects on the antioxidant-related genes Sod2, Gclc, and Hmox1, independent of their ability to clear ROS.	[87]
White and red pitaya extracts	Study of the effect of hydrolysis of artificial human gastric juice, hydrolysis of human α-amylase, and the growth of probiotics (*Lactobacillus delbrueckii* and *Bifidobacterium bifidum*)	Prebiotic properties were observed in the extracts, which included resistance to the acidic conditions of the human stomach, partial resistance to human salivary α-amylase, and the ability to stimulate the growth of lactobacilli and bifidobacteria.	[88]
Standardized cladode extracts of *Opuntia ficus-indica* and *Olea europaea*	Study conducted with one hundred healthy participants with gastrointestinal discomfort	The group treated with the extract showed a significant improvement in gastrointestinal quality of life (GIQLI) and in the Gastroesophageal Reflux Disease Symptom Rating Scale.	[89]
Fiber of *Opuntia ficus-indica*	Study carried out with 60 patients recruited with a diagnosis of irritable bowel syndrome	Dietary supplementation with fiber from *Opuntia ficus*-indica was associated with short-term improvement in symptoms of irritable bowel syndrome.	[90]

## Data Availability

The data presented in this study are available on request from the corresponding author.

## References

[1] De Albuquerque J.G., Escalona-Buendía H.B., de Aquino J.S., da Vasconcelos M.A.S. (2022). Nopal Beverage (*Opuntia ficus-indica*) as a Non-Traditional Food: Sensory Properties, Expectations, Experiences, and Emotions of Low-Income and Food-Insecure Brazilian Potential Consumers. Food Res. Int..

[2] Hernández-Becerra E., de los Angeles Aguilera-Barreiro M., Contreras-Padilla M., Pérez-Torrero E., Rodriguez-Garcia M.E. (2022). Nopal Cladodes (*Opuntia ficus indica*): Nutritional Properties and Functional Potential. J. Funct. Foods.

[3] Daniloski D., D’Cunha N.M., Speer H., McKune A.J., Alexopoulos N., Panagiotakos D.B., Petkoska A.T., Naumovski N. (2022). Recent Developments on *Opuntia* Spp., Their Bioactive Composition, Nutritional Values, and Health Effects. Food Biosci..

[4] Cardoso D.B., de Carvalho F.F.R., de Medeiros G.R., Guim A., Cabral A.M.D., Véras R.M.L., dos Santos K.C., Dantas L.C.N., de Nascimento A.G.O. (2019). Levels of Inclusion of Spineless Cactus (*Nopalea cochenillifera* Salm Dyck) in the Diet of Lambs. Anim. Feed Sci. Technol..

[5] Gusha J., Halimani T.E., Katsande S., Zvinorova P.I. (2015). The Effect of *Opuntia ficus indica* and Forage Legumes Based Diets on Goat Productivity in Smallholder Sector in Zimbabwe. Small Rumin. Res..

[6] Mayer J.A., Cushman J.C. (2019). Nutritional and Mineral Content of Prickly Pear Cactus: A Highly Water-use Efficient Forage, Fodder and Food Species. J. Agron. Crop Sci..

[7] Abbas E.Y., Ezzat M.I., El Hefnawy H.M., Abdel-Sattar E. (2022). An Overview and Update on the Chemical Composition and Potential Health Benefits of *Opuntia ficus-indica* (L.) Miller. J. Food Biochem..

[8] Angulo-Bejarano P.I., del Gómez-García M.R., Valverde M.E., Paredes-López O. (2019). Nopal (*Opuntia* Spp.) and Its Effects on Metabolic Syndrome: New Insights for the Use of a Millenary Plant. Curr. Pharm. Des..

[9] Zeghbib W., Boudjouan F., Vasconcelos V., Lopes G. (2022). Phenolic Compounds’ Occurrence in *Opuntia* Species and Their Role in the Inflammatory Process: A Review. Molecules.

[10] Cuevas-Gonzalez P.F., Liceaga A.M., Aguilar-Toala J.E. (2020). Postbiotics and Paraprobiotics: From Concepts to Applications. Food Res. Int..

[11] Koutroulis A.G. (2019). Dryland Changes under Different Levels of Global Warming. Sci. Total Environ..

[12] de Cavalcante A.M.B., Sampaio A.C.P. (2022). Modeling the Potential Distribution of Cacti under Climate Change Scenarios in the Largest Tropical Dry Forest Region in South America. J. Arid Environ..

[13] da Jardim A.M.R.F., do Araújo Júnior G.N., da Silva M.V., dos Santos A., da Silva J.L.B., Pandorfi H., de Oliveira-Júnior J.F., de Teixeira A.H.C., Teodoro P.E., de Lima J.L.M.P. (2022). Using Remote Sensing to Quantify the Joint Effects of Climate and Land Use/Land Cover Changes on the Caatinga Biome of Northeast Brazilian. Remote Sens..

[14] Dubeux J.C.B., dos Santos M.V.F., da Cunha M.V., dos Santos D.C., de Souza R.T.A., de Mello A.C.L., de Souza T.C. (2021). Cactus (*Opuntia* and *Nopalea*) Nutritive Value: A Review. Anim. Feed Sci. Technol..

[15] Abidi S., Ben Salem H., Vasta V., Priolo A. (2009). Supplementation with Barley or Spineless Cactus (*Opuntia ficus indica* f. *Inermis*) Cladodes on Digestion, Growth and Intramuscular Fatty Acid Composition in Sheep and Goats Receiving Oaten Hay. Small Rumin. Res..

[16] De Araújo F.F., de Farias D.P., Neri-Numa I.A., Pastore G.M. (2021). Underutilized Plants of the *Cactaceae* Family: Nutritional Aspects and Technological Applications. Food Chem..

[17] Ramírez-Rodríguez Y., Martínez-Huélamo M., Pedraza-Chaverri J., Ramírez V., Martínez-Tagüeña N., Trujillo J. (2020). Ethnobotanical, Nutritional and Medicinal Properties of Mexican Drylands *Cactaceae* Fruits: Recent Findings and Research Opportunities. Food Chem..

[18] Taiz L., Zeiger E., Møller I.M., Murphy A. (2017). Fisiologia e Desenvolvimento Vegetal.

[19] Messerschmid T.F.E., Wehling J., Bobon N., Kahmen A., Klak C., Los J.A., Nelson D.B., dos Santos P., de Vos J.M., Kadereit G. (2021). Carbon Isotope Composition of Plant Photosynthetic Tissues Reflects a Crassulacean Acid Metabolism (CAM) Continuum in the Majority of CAM Lineages. Perspect. Plant Ecol. Evol. Syst..

[20] Nosek M., Gawrońska K., Rozpądek P., Szechyńska-Hebda M., Kornaś A., Miszalski Z. (2018). Withdrawal from Functional Crassulacean Acid Metabolism (CAM) Is Accompanied by Changes in Both Gene Expression and Activity of Antioxidative Enzymes. J. Plant Physiol..

[21] Mabotja M.B., Gerrano A.S., Venter S.L., du Plooy C.P., Kudanga T., Amoo S.O. (2021). Nutritional Variability in 42 Cultivars of Spineless Cactus Pear Cladodes for Crop Improvement. South Afr. J. Bot..

[22] Du Toit A., de Wit M., Osthoff G., Hugo A. (2018). Antioxidant Properties of Fresh and Processed Cactus Pear Cladodes from Selected *Opuntia ficus-indica* and *O. Robusta* Cultivars. South Afr. J. Bot..

[23] Vieira É.d.A., Alcântara M.A., Albuquerque dos Santos N., Gondim A.D., Iacomini M., Mellinger C., Cordeiro A.M.T.d.M. (2021). Mucilages of Cacti from Brazilian Biodiversity: Extraction, Physicochemical and Technological Properties. Food Chem..

[24] Zhang Y., Runting R.K., Webb E.L., Edwards D.P., Carrasco L.R. (2021). Coordinated Intensification to Reconcile the ‘Zero Hunger’ and ‘Life on Land’ Sustainable Development Goals. J. Environ. Manag..

[25] Mottaleb K.A., Fatah F.A., Kruseman G., Erenstein O. (2021). Projecting Food Demand in 2030: Can Uganda Attain the Zero Hunger Goal?. Sustain. Prod. Consum..

[26] Kent K., Murray S., Penrose B., Auckland S., Godrich S., Lester E., Visentin D. (2022). Food Insecure Households Faced Greater Challenges Putting Healthy Food on the Table during the COVID-19 Pandemic in Australia. Appetite.

[27] Andreu-Coll L., Cano-Lamadrid M., Noguera-Artiaga L., Lipan L., Carbonell-Barrachina Á.A., Rocamora-Montiel B., Legua P., Hernández F., López-Lluch D. (2020). Economic Estimation of Cactus Pear Production and Its Feasibility in Spain. Trends Food Sci. Technol..

[28] Sharma P., Gaur V.K., Sirohi R., Varjani S., Hyoun Kim S., Wong J.W.C. (2021). Sustainable Processing of Food Waste for Production of Bio-Based Products for Circular Bioeconomy. Bioresour. Technol..

[29] Xu C., Nasrollahzadeh M., Selva M., Issaabadi Z., Luque R. (2019). Waste-to-Wealth: Biowaste Valorization into Valuable Bio(Nano)Materials. Chem. Soc. Rev..

[30] Da Costa G.M., Aona L.Y., Marinho L.C. (2021). Is That a Cactus on the Roof?. Cactus Succul. J..

[31] Dick M., Dal Magro L., Rodrigues R.C., de Oliveira Rios A., Flôres S.H. (2019). Valorization of *Opuntia Monacantha* (Willd.) Haw. Cladodes to Obtain a Mucilage with Hydrocolloid Features: Physicochemical and Functional Performance. Int. J. Biol. Macromol..

[32] Le Houérou H.N. (1996). The Role of Cacti (*Opuntia* spp.) in Erosion Control, Land Reclamation, Rehabilitation and Agricultural Development in the Mediterranean Basin. J. Arid Environ..

[33] Badii M.H., Flores A.E. (2001). Prickly Pear Cacti Pests and Their Control in Mexico. Fla. Entomol..

[34] Ortega-Baes P., Sühring S., Sajama J., Sotola E., Alonso-Pedano M., Bravo S., Godínez-Alvarez H. (2010). Diversity and Conservation in the Cactus Family. Desert Plants.

[35] Mufas A.H.M., Perera O.D.A.N. Study on Development of Pitaya Fruit (*Hylocereus undatus*) Incorporated Ice Cream; an Alternative Solution to the Pitaya Cultivators in Sri Lanka. Proceedings of the Third International Symposium.

[36] Ruiz-Torralba A., Guerra-Hernández E.J., García-Villanova B. (2018). Antioxidant Capacity, Polyphenol Content and Contribution to Dietary Intake of 52 Fruits Sold in Spain. CyTA J. Food.

[37] Zhen T., JingRui L., Xiang W., XiaoLei W., BinBin G., HongBo G. (2015). Cloning of Nitrate Reductase Gene of Lettuce and Effect of Exogenous γ-Aminobutyric Acid on Gene Expression and Nitrate Content in Leaves under High Nitrogen Level. Acta Bot. Boreali-Occident. Sin..

[38] Hossain F.M.d., Numan S.M.d., Akhtar S. (2021). Cultivation, Nutritional Value, and Health Benefits of Dragon Fruit (*Hylocereus* Spp.): A Review. Int. J. Hortic. Sci. Technol..

[39] Liu B., Zhang G., Murphy A., De Koeyer D., Tai H., Bizimungu B., Si H., Li X.-Q. (2016). Differences between the Bud End and Stem End of Potatoes in Dry Matter Content, Starch Granule Size, and Carbohydrate Metabolic Gene Expression at the Growing and Sprouting Stages. J. Agric. Food Chem..

[40] Gautam H., Fatma M., Sehar Z., Iqbal N., Albaqami M., Khan N.A. (2022). Exogenously-Sourced Ethylene Positively Modulates Photosynthesis, Carbohydrate Metabolism, and Antioxidant Defense to Enhance Heat Tolerance in Rice. Int. J. Mol. Sci..

[41] Mazari A., Yahiaoui K., Fedjer Z., Mahdeb A. (2018). Physical Characteristics, Phytochemical Content and Antioxidant Activity of Cactus Pear Fruits Growing in Northeast Algeria. J. Prof. Assoc. Cactus Dev..

[42] El-Beltagi H.S., Ahmed A.R., Mohamed H.I., Al-Otaibi H.H., Ramadan K.M.A., Elkatry H.O. (2023). Utilization of Prickly Pear Peels Flour as a Natural Source of Minerals, Dietary Fiber and Antioxidants: Effect on Cakes Production. Agronomy.

[43] Vargas-Solano S.V., Rodríguez-González F., Martínez-Velarde R., Campos-Mendiola R., Hurtado-Salgado M.A., Muthuswamy Ponniah J. (2022). Composición Química del Mucílago de Nopal en Diferentes Etapas de Madurez. Agrociencia.

[44] Allen J.C., Issa J.Y., Cai W. (2014). Calcium Content, in Vitro Digestibility, and Bioaccessibility in Leaves of Spinach (*Spinacia oleracea*), Sweet Potato (*Ipomea batatas*), and Drumstick Tree (*Moringa oleifera*). F1000 Res..

[45] Silva M.A., Albuquerque T.G., Pereira P., Ramalho R., Vicente F., Oliveira M.B.P.P., Costa H.S. (2021). *Opuntia ficus-indica* (L.) Mill.: A Multi-Benefit Potential to Be Exploited. Molecules.

[46] El-Mostafa K., El Kharrassi Y., Badreddine A., Andreoletti P., Vamecq J., El Kebbaj M., Latruffe N., Lizard G., Nasser B., Cherkaoui-Malki M. (2014). Nopal Cactus (*Opuntia ficus-indica*) as a Source of Bioactive Compounds for Nutrition, Health and Disease. Molecules.

[47] Khandelwal V., Mohamed M.B.N., Shukla A.K., Mangalassery S., Dayal D. (2019). Establishment and Performance of Cactus (*Opuntia ficus-indica*) Accessions at Initial Stages under Shed Net in Semi-Arid Region of Rajasthan. Int. J. Curr. Microbiol. Appl. Sci..

[48] Stambouli-Essassi S., Harrabi R., Bouzid S., Harzallah-Skhiri F. (2015). Evaluation of the Efficiency of *Opuntia ficus-indica* Cladode Cuttings for Vegetative Multiplication. Not. Bot. Horti. Agrobot. Cluj Napoca.

[49] Hernández-Urbiola M.I., Pérez-Torrero E., Rodríguez-García M.E. (2011). Chemical Analysis of Nutritional Content of Prickly Pads (*Opuntia ficus indica*) at Varied Ages in an Organic Harvest. Int. J. Environ. Res. Public Health.

[50] del Díaz M.S.S., de la Rosa A.-P.B., Héliès-Toussaint C., Guéraud F., Nègre-Salvayre A. (2017). *Opuntia* Spp.: Characterization and Benefits in Chronic Diseases. Oxid. Med Cell Longev..

[51] Madrigal-Santillán E., Portillo-Reyes J., Madrigal-Bujaidar E., Sánchez-Gutiérrez M., Izquierdo-Vega J.A., Izquierdo-Vega J., Delgado-Olivares L., Vargas-Mendoza N., Álvarez-González I., Morales-González Á. (2022). *Opuntia* Spp. in Human Health: A Comprehensive Summary on Its Pharmacological, Therapeutic and Preventive Properties. Part 2. Plants.

[52] Giraldo-Silva L., Ferreira B., Rosa E., Dias A.C.P. (2023). *Opuntia ficus-indica* Fruit: A Systematic Review of Its Phytochemicals and Pharmacological Activities. Plants.

[53] Chougui N., Sahi Y., Belkacemi M. Comparative Study between the Different Compartments of *Opuntia ficus-indica* L. Proceedings of the Inside Food Symposium.

[54] Machado F.A.P.S.A., Oliveira A.J.B., Mangolin C.A., Gobbi Filho L., Machado M.F.P.S. (2004). Polysaccharide Production from Callus Cultures of *Cereus peruvianus* Mill. (*Cactaceae*). Crop. Breed. Appl. Biotechnol..

[55] Saag L.M.K., Sanderson G.R., Moyna P., Ramos G. (1975). *Cactaceae* Mucilage Composition. J. Sci. Food Agric..

[56] Martin P., Faria-Tavares J., Mangolin C., Machado M.d.F. (2018). Somaclones of *Cereus Peruvianus* Mill. (*Cactaceae*) with High Morphological Divergence May Generate New Varieties of Ornamental Cacti and Provide Relevant Chemical Compounds. Preprints.org.

[57] Mangolin C.A., Ottoboni L.M., Machado M.F. (1999). Two-Dimensional Electrophoresis of *Cereus peruvianus* (*Cactaceae*) Callus Tissue Proteins. Electrophoresis.

[58] Som A.M., Ahmat N., Abdul Hamid H.A., Azizuddin N. (2019). A Comparative Study on Foliage and Peels of *Hylocereus undatus* (White Dragon Fruit) Regarding Their Antioxidant Activity and Phenolic Content. Heliyon.

[59] Rahimi P., Abedimanesh S., Mesbah-Namin S.A., Ostadrahimi A. (2019). Betalains, the Nature-Inspired Pigments, in Health and Diseases. Crit. Rev. Food Sci. Nutr..

[60] García-Menaya J.M., Cordobés-Durán C., Bobadilla P., Ledesma A., Pérez-Rangel I. (2009). Hypersensitivity Systemic Reaction to Cactus Fruit (*Opuntia ficus-indica*). Allergy.

[61] Liguori G., Gaglio R., Greco G., Gentile C., Settanni L., Inglese P. (2021). Effect of *Opuntia ficus-indica* Mucilage Edible Coating on Quality, Nutraceutical, and Sensorial Parameters of Minimally Processed Cactus Pear Fruits. Agronomy.

[62] Saparbekova A., Latif A., Altekey A. (2021). Risks of Microbiological Contamination of Fruits and Vegetables Used for Food. Bull. Innov. Univ. Eurasia.

[63] Rodrigues C., de Paula C.D., Lahbouki S., Meddich A., Outzourhit A., Rashad M., Pari L., Coelhoso I., Fernando A.L., Souza V.G.L. (2023). *Opuntia* Spp.: An Overview of the Bioactive Profile and Food Applications of This Versatile Crop Adapted to Arid Lands. Foods.

[64] Cruz-Rubio J.M., Mueller M., Viernstein H., Loeppert R., Praznik W. (2021). Prebiotic Potential and Chemical Characterization of the Poly and Oligosaccharides Present in the Mucilage of *Opuntia ficus-indica* and *Opuntia joconostle*. Food Chem..

[65] Guevara-Arauza J.C., de Jesús Ornelas-Paz J., Pimentel-González D.J., Rosales Mendoza S., Soria Guerra R.E., Paz Maldonado L.M.T. (2012). Prebiotic Effect of Mucilage and Pectic-Derived Oligosaccharides from Nopal (*Opuntia ficus-indica*). Food Sci. Biotechnol..

[66] Cruz-Rubio J.M., Mueller M., Loeppert R., Viernstein H., Praznik W. (2020). The Effect of Cladode Drying Techniques on the Prebiotic Potential and Molecular Characteristics of the Mucilage Extracted from *Opuntia ficus-indica* and *Opuntia joconostle*. Sci. Pharm..

[67] Ribeiro T.S., Sampaio K.B., Menezes F.N.D.D., de Assis P.O.A., dos Santos Lima M., de Oliveira M.E.G., de Souza E.L., de Cássia Ramos do Egypto Queiroga R. (2020). In Vitro Evaluation of Potential Prebiotic Effects of a Freeze-Dried Juice from Pilosocereus Gounellei (A. Weber Ex K. Schum. Bly. Ex Rowl) Cladodes, an Unconventional Edible Plant from Caatinga Biome. 3 Biotech.

[68] Reyes-Reyes M., Salazar-Montoya J.A., Rodríguez-Páez L.I., Ramos-Ramírez E.G. (2019). In Vitro Fermentation of Oligosaccharides Obtained from Enzymatic Hydrolysis of *Opuntia streptacantha* Mucilage. J. Sci. Food Agric..

[69] Peña-Valdivia C.B., Trejo C., Arroyo-Peña V.B., Sánchez Urdaneta A.B., Balois Morales R. (2012). Diversity of Unavailable Polysaccharides and Dietary Fiber in Domesticated Nopalito and Cactus Pear Fruit (*Opuntia* Spp.). Chem. Biodivers..

[70] Hussain S., Alamri M.S., Mohamed A.A., Ibraheem M.A., Qasem A.A.A., Shamlan G., Ababtain I.A. (2022). Exploring the Role of Acacia (*Acacia seyal*) and Cactus (*Opuntia ficus-indica*) Gums on the Dough Performance and Quality Attributes of Breads and Cakes. Foods.

[71] Uebelhack R., Busch R., Alt F., Beah Z.-M., Chong P.-W. (2014). Effects of Cactus Fiber on the Excretion of Dietary Fat in Healthy Subjects: A Double Blind, Randomized, Placebo-Controlled, Crossover Clinical Investigation. Curr. Ther. Res..

[72] Sirotkin A.V. (2022). Can Nopal Cactus (*Opuntia ficus-indica* L. Miller) Treat Obesity?. Obes. Med.

[73] Ayuso M., Carpena M., Taofiq O., Albuquerque T.G., Simal-Gandara J., Oliveira M.B.P.P., Prieto M.A., Ferreira I.C.F.R., Barros L. (2022). Fig “*Ficus Carica* L.” and Its by-Products: A Decade Evidence of Their Health-Promoting Benefits towards the Development of Novel Food Formulations. Trends Food Sci. Technol..

[74] Feugang J.M. (2006). Nutritional and Medicinal Use of Cactus Pear (*Opuntia* Spp.) Cladodes and Fruits. Front. Biosci..

[75] Chandel V., Biswas D., Roy S., Vaidya D., Verma A., Gupta A. (2022). Current Advancements in Pectin: Extraction, Properties and Multifunctional Applications. Foods.

[76] Goycoolea F.M., Cárdenas A. (2003). Pectins from *Opuntia* Spp.: A Short Review. J. Prof. Assoc. Cactus Dev..

[77] Costa K.P.B., Reichembach L.H., de Oliveira Petkowicz C.L. (2022). Pectins with Commercial Features and Gelling Ability from Peels of *Hylocereus* Spp. Food Hydrocoll..

[78] Cárdenas A., Goycoolea F.M., Rinaudo M. (2008). On the Gelling Behaviour of ‘Nopal’ (*Opuntia ficus indica*) Low Methoxyl Pectin. Carbohydr. Polym..

[79] Mohamed S.K., Alazhary A.M., Al-Zaqri N., Alsalme A., Alharthi F.A., Hamdy M.S. (2020). Cost-Effective Adsorbent from Arabinogalactan and Pectin of Cactus Pear Peels: Kinetics and Thermodynamics Studies. Int. J. Biol. Macromol..

[80] Blanco-Pérez F., Steigerwald H., Schülke S., Vieths S., Toda M., Scheurer S. (2021). The Dietary Fiber Pectin: Health Benefits and Potential for the Treatment of Allergies by Modulation of Gut Microbiota. Curr. Allergy Asthma Rep..

[81] Liu Z., Zhang J., Zhao Q., Wen A., Li L., Zhang Y. (2022). The Regulating Effect of Tibet *Opuntia ficus-indica* (Linn.) Mill. Polysaccharides on the Intestinal Flora of Cyclophosphamide-Induced Immunocompromised Mice. Int. J. Biol. Macromol..

[82] Corona-Cervantes K., Parra-Carriedo A., Hernández-Quiroz F., Martínez-Castro N., Vélez-Ixta J.M., Guajardo-López D., García-Mena J., Hernández-Guerrero C. (2022). Physical and Dietary Intervention with *Opuntia ficus-indica* (Nopal) in Women with Obesity Improves Health Condition through Gut Microbiota Adjustment. Nutrients.

[83] Sánchez-Tapia M., Aguilar-López M., Pérez-Cruz C., Pichardo-Ontiveros E., Wang M., Donovan S.M., Tovar A.R., Torres N. (2017). Nopal (*Opuntia ficus indica*) Protects from Metabolic Endotoxemia by Modifying Gut Microbiota in Obese Rats Fed High Fat/Sucrose Diet. Sci. Rep..

[84] Song H., Chu Q., Yan F., Yang Y., Han W., Zheng X. (2016). Red Pitaya Betacyanins Protects from Diet-Induced Obesity, Liver Steatosis and Insulin Resistance in Association with Modulation of Gut Microbiota in Mice. J. Gastroenterol. Hepatol..

[85] Ali S.K., Mahmoud S.M., El-Masry S.S., Alkhalifah D.H.M., Hozzein W.N., Aboel-Ainin M.A. (2022). Phytochemical Screening and Characterization of the Antioxidant, Anti-Proliferative and Antibacterial Effects of Different Extracts of *Opuntia ficus-indica* Peel. J. King Saud. Univ. Sci..

[86] Rockett F.C., de Oliveira Schmidt H.O., Schmidt L., Rodrigues E., Tischer B., Ruffo de Oliveira V., Lima da Silva V., Rossini Augusti P., Flôres S.H., Rios A. (2020). Phenolic Compounds and Antioxidant Activity in Vitro and in Vivo of Butia and *Opuntia* Fruits. Food Res. Int..

[87] González-Ponce H.A., Martínez-Saldaña M.C., Tepper P.G., Quax W.J., Buist-Homan M., Faber K.N., Moshage H. (2020). Betacyanins, Major Components in *Opuntia* Red-Purple Fruits, Protect against Acetaminophen-Induced Acute Liver Failure. Food Res. Int..

[88] Wichienchot S., Jatupornpipat M., Rastall R.A. (2010). Oligosaccharides of Pitaya (Dragon Fruit) Flesh and Their Prebiotic Properties. Food Chem..

[89] Malfa G.A., Di Giacomo C., Cardia L., Sorbara E.E., Mannucci C., Calapai G. (2021). A Standardized Extract of *Opuntia ficus-indica* (L.) Mill and *Olea europaea* L. Improves Gastrointestinal Discomfort: A double-blinded Randomized-controlled Study. Phytother. Res..

[90] Remes-Troche J.M., Taboada-Liceaga H., Gill S., Amieva-Balmori M., Rossi M., Hernández-Ramírez G., García-Mazcorro J.F., Whelan K. (2021). Nopal Fiber (*Opuntia ficus*-*indica*) Improves Symptoms in Irritable Bowel Syndrome in the Short Term: A Randomized Controlled Trial. Neurogastroenterol. Motil..

[91] Montiel-Sánchez M., García-Cayuela T., Gómez-Maqueo A., García H.S., Cano M.P. (2021). In Vitro Gastrointestinal Stability, Bioaccessibility and Potential Biological Activities of Betalains and Phenolic Compounds in Cactus Berry Fruits (*Myrtillocactus geometrizans*). Food Chem..

[92] Gómez-Maqueo A., García-Cayuela T., Fernández-López R., Welti-Chanes J., Cano M.P. (2019). Inhibitory Potential of Prickly Pears and Their Isolated Bioactives against Digestive Enzymes Linked to Type 2 Diabetes and Inflammatory Response. J. Sci. Food Agric..

[93] del Laguna B.C.C., Flores Gallegos A.C., Ascacio Valdés J.A., Iliná A., Galindo A.S., Castañeda Facio A.O., Esparza González S.C., Herrera R.R. (2022). Physicochemical and Functional Properties of the Undervalued Fruits of Cactus Cylindropuntia Imbricate (“Xoconostle”) and Antioxidant Potential. Biocatal. Agric. Biotechnol..

[94] El-Hawary S.S., Sobeh M., Badr W.K., Abdelfattah M.A.O., Ali Z.Y., El-Tantawy M.E., Rabeh M.A., Wink M. (2020). HPLC-PDA-MS/MS Profiling of Secondary Metabolites from *Opuntia ficus-indica* Cladode, Peel and Fruit Pulp Extracts and Their Antioxidant, Neuroprotective Effect in Rats with Aluminum Chloride Induced Neurotoxicity. Saudi J. Biol. Sci..

[95] Rocchetti G., Pellizzoni M., Montesano D., Lucini L. (2018). Italian *Opuntia ficus-indica* Cladodes as Rich Source of Bioactive Compounds with Health-Promoting Properties. Foods.

[96] El Otmani S., Chentouf M., Hornick J.L., Cabaraux J.F. (2019). Chemical Composition and in Vitro Digestibility of Alternative Feed Resources for Ruminants in Mediterranean Climates: Olive Cake and Cactus Cladodes. J. Agric. Sci..

[97] Dick M., Limberger C., Cruz Silveira Thys R., de Oliveira Rios A., Hickmann Flôres S. (2020). Mucilage and Cladode Flour from Cactus (*Opuntia monacantha*) as Alternative Ingredients in Gluten-Free Crackers. Food Chem..

[98] De Santiago E., Juániz I., Cid C., De Peña M.-P. (2021). Extraction of (Poly)Phenolic Compounds of Cactus (*Opuntia ficus-indica* (L.) Mill.) Cladodes. Food Anal. Methods.

[99] Shen N., Wang T., Gan Q., Liu S., Wang L., Jin B. (2022). Plant Flavonoids: Classification, Distribution, Biosynthesis, and Antioxidant Activity. Food Chem..

[100] Matboli M., Saad M., Hasanin A.H., Saleh L.A., Baher W., Bekhet M.M., Eissa S. (2021). New Insight into the Role of Isorhamnetin as a Regulator of Insulin Signaling Pathway in Type 2 Diabetes Mellitus Rat Model: Molecular and Computational Approach. Biomed. Pharmacother..

[101] Gong G., Guan Y.-Y., Zhang Z.-L., Rahman K., Wang S.-J., Zhou S., Luan X., Zhang H. (2020). Isorhamnetin: A Review of Pharmacological Effects. Biomed. Pharmacother..

[102] Al-Naqeb G., Fiori L., Ciolli M., Aprea E. (2021). Prickly Pear Seed Oil Extraction, Chemical Characterization and Potential Health Benefits. Molecules.

[103] De Wit M., Motsamai V.K., Hugo A. (2021). Cold-Pressed Cactus Pear Seed Oil: Quality and Stability. Grasas Aceites.

[104] Nounah I., Chbani M., Matthäus B., Charrouf Z., Hajib A., Willenberg I. (2020). Profile of Volatile Aroma-Active Compounds of Cactus Seed Oil (*Opuntia ficus-indica*) from Different Locations in Morocco and Their Fate during Seed Roasting. Foods.

[105] Al-Naqeb G., Cafarella C., Aprea E., Ferrentino G., Gasparini A., Buzzanca C., Micalizzi G., Dugo P., Mondello L., Rigano F. (2023). Supercritical Fluid Extraction of Oils from Cactus *Opuntia ficus-indica* L. and *Opuntia Dillenii* Seeds. Foods.

[106] Da Silva Dantas D.L., Viera V.B., Soares J.K.B., dos Santos K.M.O., do Egito A.S., de Figueirêdo R.M.F., Lima M.d.S., Machado N.A.F., de Souza M.d.F.V., da Conceição M.L. (2022). *Pilosocereus gounellei* (Xique-Xique) Flour: Improving the Nutritional, Bioactive, and Technological Properties of Probiotic Goat-Milk Yogurt. LWT.

[107] Machado T.A.D.G., Pacheco M.T.B., Queiroga R.d.C.R.d.E., Cavalcante L.M., Bezerril F.F., Ormenese R.d.C.S.C., Garcia A.d.O., Nabeshima E.H., Pintado M.M.E., Oliveira H.M.L. (2021). Nutritional, Physicochemical and Sensorial Acceptance of Functional Cookies Enriched with Xiquexique (*Pilosocereus gounellei*) Flour. PLoS ONE.

[108] Bouazizi S., Montevecchi G., Antonelli A., Hamdi M. (2020). Effects of Prickly Pear (*Opuntia ficus-indica* L.) Peel Flour as an Innovative Ingredient in Biscuits Formulation. LWT.

[109] Msaddak L., Abdelhedi O., Kridene A., Rateb M., Belbahri L., Ammar E., Nasri M., Zouari N. (2017). *Opuntia ficus-indica* Cladodes as a Functional Ingredient: Bioactive Compounds Profile and Their Effect on Antioxidant Quality of Bread. Lipids Health Dis..

[110] Ali R.F.M., El-Anany A.M., Mousa H.M., Hamad E.M. (2020). Nutritional and Sensory Characteristics of Bread Enriched with Roasted Prickly Pear (*Opuntia ficus-indica*) Seed Flour. Food Funct..

[111] Huang J., Hu Z., Li G., Hu L., Chen J., Hu Y. (2022). Make Your Packaging Colorful and Multifunctional: The Molecular Interaction and Properties Characterization of Natural Colorant-Based Films and Their Applications in Food Industry. Trends Food Sci. Technol..

[112] Utpott M., Assis R.Q., Pagno C.H., Pereira Krigger S., Rodrigues E., de Oliveira Rios A., Hickmann Flôres S. (2020). Evaluation of the Use of Industrial Wastes on the Encapsulation of Betalains Extracted from Red Pitaya Pulp (*Hylocereus polyrhizus*) by Spray Drying: Powder Stability and Application. Food Bioproc. Tech..

[113] Carmona J.C., Robert P., Vergara C., Sáenz C. (2021). Microparticles of Yellow-Orange Cactus Pear Pulp (*Opuntia ficus-indica*) with Cladode Mucilage and Maltodextrin as a Food Coloring in Yogurt. LWT.

[114] Ruiz-Gutiérrez M.G., Amaya-Guerra C.A., Quintero-Ramos A., Pérez-Carrillo E., Meléndez-Pizarro C.O. (2017). Use of Red Cactus Pear (*Opuntia ficus-indica*) Encapsulated Powder to Pigment Extruded Cereal. J. Food Qual..

[115] Lugo-Zarate L., del Cruz-Cansino N.S., Ramírez-Moreno E., Zafra-Rojas Q.Y., Calderón-Ramos Z.G., Delgado-Olivares L., Arias-Rico J., Cervantes-Elizarrarás A. (2021). Evaluation of Physicochemical, Microbiological, and Antioxidant Properties of a Drinkable Yogurt Added with Ultrasonicated Purple Cactus Pear (*Opuntia ficus-indica*) Juice Powder. J. Food Process. Preserv..

[116] Da Nunes A.R.C., Mangolin C.A., Braz de Oliveira A.J., Gonçalves R.A.C., da Avincola A.S., Ribeiro de Almeida R.T., Pilau E.J., de Fatima Pires da Silva Machado M. (2022). *Cereus peruvianus* Mill. (*Cactaceae*) as a Source of Natural Antioxidants: Phenolic Compounds and Antioxidant Activity of Cladode Extracts in Two Collection Periods. Curr. Res. Food Sci..

[117] Oliveira J.M.C., de Souza E.L., de Lima K.Y.G., dos Lima M.S., Viera V.B., Queiroga R.d.C.R.d.E., de Oliveira M.E.G. (2021). Physicochemical Parameters, Phytochemical Profile and Antioxidant Properties of a New Beverage Formulated with Xique-Xique (*Pilosocereus gounellei*) Cladode Juice. Foods.

[118] Moussa-Ayoub T.E., Jäger H., Knorr D., El-Samahy S.K., Kroh L.W., Rohn S. (2017). Impact of Pulsed Electric Fields, High Hydrostatic Pressure, and Thermal Pasteurization on Selected Characteristics of Opuntia Dillenii Cactus Juice. LWT Food Sci. Technol..

[119] Otálora M.C., de Jesús Barbosa H., Perilla J.E., Osorio C., Nazareno M.A. (2019). Encapsulated Betalains (*Opuntia ficus-indica*) as Natural Colorants. Case Study: Gummy Candies. LWT.

[120] Moussa-Ayoub T.E., Youssef K., El-Samahy S.K., Kroh L.W., Rohn S. (2015). Flavonol Profile of Cactus Fruits (*Opuntia ficus-indica*) Enriched Cereal-Based Extrudates: Authenticity and Impact of Extrusion. Food Res. Int..

[121] Tabarestani P.S., Kashiri M., Maghsoudlou Y., Shahiri Tabarestani H., Ghorbani M. (2022). Effect of *Opuntia* Pulp as a Clean Label Ingredient on Techno-functional Properties of Meat-free Burger. Int. J. Food Sci. Technol..

[122] Panda S.K., Behera S.K., Witness Qaku X., Sekar S., Ndinteh D.T., Nanjundaswamy H.M., Ray R.C., Kayitesi E. (2017). Quality Enhancement of Prickly Pears (*Opuntia* Sp.) Juice through Probiotic Fermentation Using Lactobacillus Fermentum—ATCC 9338. LWT.

[123] Verón H.E., Gauffin Cano P., Fabersani E., Sanz Y., Isla M.I., Fernández Espinar M.T., Gil Ponce J.V., Torres S. (2019). Cactus Pear (*Opuntia ficus-indica*) Juice Fermented with Autochthonous *Lactobacillus plantarum* S-811. Food Funct..

[124] Stintzing F.C., Schieber A., Carle R. (2001). Phytochemical and Nutritional Significance of Cactus Pear. Eur. Food Res. Technol..

[125] Alcántara-Zavala A.E., de Dios Figueroa-Cárdenas J. (2022). Shelf Life, Physicochemical and Antioxidant Properties of Red Cactus Pear Pulque Processed by Ohmic Heating and by Conventional Pasteurization. Int. J. Gastron. Food Sci..

[126] El-Sayed H., Ramadan M. (2020). Production of Probiotic-Fermented Rice Milk Beverage Fortified with Cactus Pear and *Physalis* Pulp. Zagazig J. Agric. Res..

[127] Hou L., Kou X., Wang H., Liu Y., Xie B., Yang X. (2013). Study on the Processing Technology of Compound Fruit and Vegetable Beverage of Cactus. Storage Process.

[128] Tsegay Z.T., Gebremedhin K.M. (2019). Physicochemical and Sensory Properties of Wine Produced from Blended Cactus Pear (*Opuntia ficus-indica*) and *Lantana camara* (*L. camara* ) Fruits. J. Food Qual..

[129] Ayed L., Hamdi M. (2015). Manufacture of a Beverage from Cactus Pear Juice Using “Tea Fungus” Fermentation. Ann. Microbiol..

